# Hospital outpatient perceptions of the physical environment of waiting areas: the role of patient characteristics on atmospherics in one academic medical center

**DOI:** 10.1186/1472-6963-7-198

**Published:** 2007-12-05

**Authors:** Chun-Yen Tsai, Mu-Chia Wang, Wei-Tsen Liao, Jui-Heng Lu, Pi-hung Sun, Blossom Yen-Ju Lin, Gerald-Mark Breen

**Affiliations:** 1 Institute of Health Service Administration, China Medical University, Taiwan; 2 Department of Business Administration, San Francisco State University, USA; 3 Department of Public Affairs, University of Central Florida, USA

## Abstract

**Background:**

This study examines hospital outpatient perceptions of the physical environment of the outpatient waiting areas in one medical center. The relationship of patient characteristics and their perceptions and needs for the outpatient waiting areas are also examined.

**Method:**

The examined medical center consists of five main buildings which house seventeen primary waiting areas for the outpatient clinics of nine medical specialties: 1) Internal Medicine; 2) Surgery; 3) Ophthalmology; 4) Obstetrics-Gynecology and Pediatrics; 5) Chinese Medicine; 6) Otolaryngology; 7) Orthopedics; 8) Family Medicine; and 9) Dermatology. A 15-item structured questionnaire was developed to rate patient satisfaction covering the four dimensions of the physical environments of the outpatient waiting areas: 1) visual environment; 2) hearing environment; 3) body contact environment; and 4) cleanliness. The survey was conducted between November 28, 2005 and December 8, 2005. A total of 680 outpatients responded. Descriptive, univariate, and multiple regression analyses were applied in this study.

**Results:**

All of the 15 items were ranked as relatively high with a range from 3.362 to 4.010, with a neutral score of 3. Using a principal component analysis' summated scores of four constructed dimensions of patient satisfaction with the physical environments (i.e. visual environment, hearing environment, body contact environment, and cleanliness), multiple regression analyses revealed that patient satisfaction with the physical environment of outpatient waiting areas was associated with gender, age, visiting frequency, and visiting time.

**Conclusion:**

Patients' socio-demographics and context backgrounds demonstrated to have effects on their satisfaction with the physical environment of outpatient waiting areas. In addition to noticing the overall rankings for less satisfactory items, what should receive further attention is the consideration of the patients' personal characteristics when redesigning more comfortable and customized physical environments of waiting areas.

## Background

Kotler [1] first introduced the concept of "atmospherics," a term that refers to how the physical and controllable components of an environment affect a buyer's "purchasing propensity." Other marketing professionals have also pointed out that the use of atmospherics can lead to customer satisfaction, patronage, and advertising via word-of-mouth [2-7]. From the customer's perspective, atmospherics involves much more than the design and construction of the physical surroundings. This concept implies and encompasses the cognitive, emotional, and physiological influences on customers [8]. Several previous studies have explored the physical environments in healthcare settings. For example, Woodside et al. [9] found that location, equipment, and facility were important factors that hospital patients sought to optimize. For dental offices [10], organization, neatness, comfort of seating, magazine selection, and music all had a significant impact on dental service satisfaction [11]. Gotlieb [12] found that patients' perceptions of their hospital rooms could influence patients' perception of hospital quality. Participants in 16 focus groups in four major cities in the U.S.A. (that is, Baltimore, Los Angeles, Phoenix, and Orlando) identified that cleanliness of the hospital rooms and bathrooms were one of the most noted items for quality of hospital care [13]. Akinci *et al.*[14] reported that outpatients in four Turkish hospitals indicated that the physical appearance of the hospital is a significant factor in the hospital selection process. Further, Douglas and Douglas [15] surveyed inpatients and noted that aspects such as transportation, ground and landscape design, as well as space planning, were also important factors in the hospital selection process.

Previous studies have explored methods to improve service quality in outpatient departments by analyzing outpatient satisfaction regarding waiting times [16-22], courtesy and interpersonal skills [17,20,21,23,24], professionalism [17], access [23,24], patient preferences and expectations [21,23], coordination of care [21,23], education and information provision [16,20,23,24], emotional support [23], technical quality of care [17], and overall quality and satisfaction [23]. The idea to design outpatient departments based on the opinions of patients was derived from the results of two outpatient satisfaction questionnaires in Greece [25] and France [26]. The items in the questionnaires related to aspects of outpatient hospitals, including attractiveness and size, cleanliness, ease in finding a seat to wait for a physician, room temperature, and the conditions of the bathrooms in the waiting areas. Cho *et al.*[27] examined the relationship between service quality and outpatient satisfaction in a Korean general hospital. They queried patient satisfaction with tangible elements in waiting rooms as indicators of service quality, such as the pleasantness of waiting areas, the ease of using amenities, the quality and newness of the equipment, and the ease in locating care facilities. The researchers found that the perceived quality of tangible environments by patients who visited more than six times was positively related to patient satisfaction.

In Taiwan, there are no specific rules regarding healthcare providers in any official documents and facility accreditation relating to the design of their outpatient waiting areas. From a marketing perspective, however, the concept of "atmospherics" has pervaded the provider side and has been viewed as a method to provide the customers (that is, patients and visitors) with more friendly and humane healing environments to attract patients, while giving them the freedom to choose their preferred healthcare providers. The vision of this study was originally aimed to raise the issue of physical environments in the healthcare industry, not just from architectural or interior design perspectives, but also from the users' (patient) perspective. In this study, we examined the physical environments of various outpatient waiting areas of a medical center that has the largest annual volume of outpatients in central Taiwan. In addition, several researchers have found that patient characteristics, including age [17,28-32], education [28,30-32] and gender [28,30-32], were independent predictors of patient satisfaction. We also explored how patient characteristics might be associated with their perceptions of waiting areas in outpatient departments. From the marketing management perspective, these results may help hospital administrators understand how to effectively construct physical environments that are more convenient and comfortable for patients.

## Methods

### Background of outpatient waiting areas in the studied medical center

The studied medical center is a 1,702-bed institution located in central Taiwan. The medical center employs a total of 3,609 staff. The average monthly volume of outpatients was 140,040 between 2001 to 2005 [33].

The medical center comprises five buildings that house seventeen outpatient waiting areas for nine medical specialties: 1) four waiting rooms for Internal Medicine; 2) three for Surgery; 3) one for Ophthalmology; 4) two for Obstetrics-Gynecology and Pediatrics; 5) three for Chinese Medicine; 6) one for Otolaryngology; 7) one for Orthopedics; 8) one for Family Medicine; and 9) one for Dermatology.

### Study sample and data collection

Data for this study were collected between November 28, 2005 to December 8, 2005, a time period in which there were not any special holidays in Taiwan that could create a possible bias from the use of seasonal decorations in outpatient waiting areas. The surveyed patients were randomly selected from the 17 individual waiting areas, from nine o'clock in the morning to four o'clock in the afternoon, Monday to Friday, during the one-week study period, to capture all time stages of outpatient visits. The five trained researchers distributed the questionnaires to the sampled patients and explained the purpose of the survey. Informed consent was obtained from each participant in the study. All the survey items were completed by either the outpatients or their guardians; guardians were used if the sampled patients had difficulty reading or writing. In addition, the researchers explained the meanings of the questions when the respondents could not understand the survey items, and the researchers also verified the questionnaires for completeness when the respondents submitted their surveys. Incomplete surveys were returned to the respondents for completion. Patient personal background information (the respondents) was obtained; however, disclosure of monthly income was not requested or required, as seeking this information is considered inappropriate, private, and sensitive in the Taiwanese culture. Overall, the rejection rate in the surveying process was approximately 10%, which, for Taiwanese people, is a relatively low percentage; the researchers wore student IDs as a means to increase trust from the public and respondents. Finally, a total of 680 patients, or 40 patients from each of the 17 waiting areas, completed the survey and were included in this study.

### Study instruments

A 15-item structured questionnaire was developed to rate patient satisfaction of the dimensions of the physical environments of the outpatient waiting areas, based on four human senses: sight, sound, smell, and touch [8,34]. In addition, the restrooms in the waiting areas were also examined. The structured questionnaire was developed with the wording of practical managerial actions, including lighting, ground and landscape design, furniture layouts, color design, space design, noise level, volume of paging and broadcast services, air freshness, room temperature, seating comfort and sufficiency, and cleanliness. For the patient perceptions of the various physical environments in this study, items were scored on a 5-point Likert scale ranging from 1 to 5 (1 = strongly dissatisfied, 2 = dissatisfied, 3 = fair, 4 = satisfied, and 5 = strongly satisfied). A question item was recorded as "not applicable" when the respondent had no experience with an item. The detailed information of the individual item questions is listed in Table 1.

**Table 1:** Principal component analysis for 15-item physical environment evaluation of outpatient waiting areas

**Physical environment of outpatient waiting areas**	**Component 1: Visual environment**	**Component 2: Hearing environment**	**Component 3: Body contact environment**	**Component 4: Cleanliness (overall and restrooms)**
1. Lighting	0.636			
2. Ground and landscape design	0.846			
3. Furniture layouts	0.813			
4. Color design	0.698			
5. Space design	0.500			
6. Noise level		0.592		
7. Volume of paging		0.843		
8. Volume of broadcast services		0.817		
9. Air freshness			0.442	
10. Temperature			0.475	
11. Seating comfort			0.719	
12. Seating sufficiency			0.815	
13. Cleanliness				0.510
14. Air freshness of restrooms				0.860
15. Cleanliness of restrooms				0.861

Note: Rotation method: Varimax with Kaiser Normalization

The structured questionnaires were first drafted and then examined by two academic professors and two hospital administrators for theoretical accuracy. Then, one pilot study was pre-tested for 25 patients. The wordings and meanings of each question item were revised to ensure content validity.

The questionnaire also covered questions about the possible need for other ancillaries to improve the overall physical environments, including a wall-mounted television, newspapers, health education brochures, water, and access to wheel chairs. Demographic information, including gender, age, education, living location, and monthly incomes, was also colleted. Data regarding the number of prior visits, the time of day the patient visited (morning or afternoon), and the specialty departments were recorded.

### Analytical techniques

The data were first analyzed descriptively by computing means and standard deviations for continuous variables, and frequencies and percentages for categorical variables. Several researchers have discussed the potential pitfalls in using the individual single item for psychological attributes; that is, individual items have considerable random measurement error, individual items can only categorize the respondents into relatively small numbers of groups, individual items lack scopes, and a single item is very unlikely to fully represent a complex theoretical concept or any attributes [35-37]. Therefore, the summated scores would be better indices to employ in this study of outpatients' perception to the physical environment in the waiting areas. Principal component analysis was performed for the 15 individual items at a significance level 0.05. Four summated indices from the 15 question items of physical environments were extracted as "visual environment", "hearing environment", "body contact environment", and "cleanliness" (see Table 1), which explains the total variance of 65% (Kaiser-Meyer-Olkin of sampling adequacy = 0.893 and Bartlett's test of Sphericity = 0.00).

The univariate analyses including ANOVA, t-test, simple regression, and multiple regression analyses were conducted to examine the relationship between patients' personal and contextual characteristics and four summated indices of the physical environments in the waiting areas [32].

## Results

### Outpatient personal and contextual characteristics

Of the 680 respondents, 54.3% (n = 369) self-answered the questionnaire. Females comprised 59% of the respondents and ranged in age from 16 to 86 years (mean = 37.56 years). Most respondents had undergraduate degrees or above (54.7%), were city residents (54.7%), and were first-time outpatients at this medical center (81.3%). Half of the respondents visited in the morning and half visited in the afternoon (see Table 2).

**Table 2:** Background information of the respondents in the study of outpatient waiting areas (n = 680)

**Variables**	**Scale**	**Frequency**	**%**	**Mean**	**SD**
***Patient characteristics***					
**Gender**	Male	277	40.70		
	Female	403	59.30		
**Age (years)**		Min:16	Max: 86	37.56	12.84
**Education**	Undergraduate and above	372	54.71		
	Senior high school	213	31.32		
	Junior high school	36	5.29		
	Elementary school and below	21	3.09		
	Missing	38	5.59		
**Monthly income (money rate: Taiwan NT$: USA$ ≈ 33:1)**					
	NT$20,000 below	67	9.85		
	NT$20,000–29,999	124	18.24		
	NT$30,000–49,999	157	23.09		
	NT$50,000–69,999	83	12.21		
	NT$70,000–above	45	6.62		
	No salary	104	15.29		
	Missing	100	14.71		
**Living area**	City residents	372	54.71		
	Outside city residents	276	40.59		
	Missing	32	4.71		
***Patient visiting information***					
**Visits**	First-time patients	553	0.81		
	Returning patients	124	0.18		
	Missing	3	0.00		
**Visiting time**	Morning	340	50.0		
	Afternoon	340	50.0		
**Waiting areas**	Internal Medicines	160	23.53		
	Surgeries	120	17.65		
	Ophthalmology	40	5.88		
	Obstetrics-Gynecology-Pediatrics	80	11.76		
	Chinese Medicine	120	17.65		
	Otolaryngology	40	5.88		
	Orthopedics	40	5.88		
	Family Medicine	40	5.88		
	Dermatology	40	5.88		

### Patient satisfaction with the physical environments of waiting areas: Analysis of 15 individual items

Among the dimensions of the physical environment evaluated by outpatients, cleanliness of the waiting areas (mean = 4.010) was ranked as the most satisfactory dimension, followed by lighting (mean = 3.895) and cleanliness of restrooms (mean = 3.808). Noise level was the least satisfactory dimension in the waiting areas, whereas the number and comfort of chairs in the waiting areas were ranked as the bottom three. Patients were most dissatisfied with the number of chairs and chair comfort as well as the noise level. Patients were also dissatisfied with the temperature of the waiting areas, that is, the high percentage of temperature dissatisfaction (6.6%) in the waiting areas; although the mean was not ranked as low (see Table 3).

**Table 3:** Descriptive analyses of physical environments in the outpatient waiting areas

**Physical environment of outpatient waiting areas**				**Frequency (%)**					
				
	**Mean**	**SD**	**Ranking+**	**Very dissatisfied (Likert 1)**	**Dissatisfied (Likert 2)**	**Fair (Likert 3)**	**Satisfied (Likert 4)**	**Very satisfied (Likert 5)**	**Not applicable**
1. Lighting	3.895	0.687	2	0.29	1.62	22.65	58.97	16.32	0.15
2. Ground and landscape design	3.664	0.728	8	0.29	2.94	38.24	47.06	11.47	0.00
3. Furniture layouts	3.604	0.706	10	0.29	3.53	39.85	47.35	8.53	0.44
4. Color design	3.753	0.723	4	0.00	3.97	29.41	53.53	12.79	0.29
5. Space design	3.593	0.760	11	0.29	6.32	36.32	46.91	9.41	0.74
6. Noise level	3.362	0.802	15	0.88	10.59	47.50	33.68	7.35	0.00
7. Volume of paging	3.731	0.703	5	0.44	2.94	29.85	55.44	10.44	0.88
8. Volume of broadcast services	3.719	0.674	7	0.29	2.06	31.18	53.68	9.26	3.53
9. Air freshness	3.580	0.797	12	0.88	7.79	32.50	49.26	8.97	0.59
10. Temperature	3.724	0.774	6	0.44	6.18	26.18	54.56	12.35	0.29
11. Seating comfort	3.398	0.821	14	1.32	10.88	40.88	39.56	6.76	0.44
12. Seating sufficiency	3.427	0.895	13	1.91	13.09	33.97	41.76	8.82	0.44
13. Cleanliness	4.010	0.641	1	0.00	0.74	17.79	61.03	20.29	0.15
14. Air freshness of restrooms	3.647	0.759	9	0.44	5.00	30.29	46.03	9.56	8.68
15. Cleanliness of restrooms	3.808	0.745	3	0.44	3.38	23.09	51.18	13.68	8.24

Note: + Lower number was shown as higher satisfaction by comparison of mean values for individual question items of physical environments

### Patient satisfaction indices of physical environment in the outpatient waiting areas

The principal component analysis performed to test the construct validity revealed that the scale was loaded by four components. The first component titled "visual environment" was loaded by the five items: lighting, ground and landscape design, furniture layouts, color design, and space design. The second component titled "hearing environment" was loaded by the three items: noise level, volume of paging, and broadcast services. The third component titled "body contact environment" was loaded by the four items: air freshness, temperature, seating comfort, and sufficiency. And the fourth component titled "cleanliness" was loaded by the three items: holistic cleanliness, and cleanliness and air freshness of restrooms (see Table 1). Internal consistency measured as the Cronbach α value for these four summated indices of the outpatient physical environment; visual environment, hearing environment, body contact environment, and cleanliness were 0.839, 0.746, 0.756, and 0.799, respectively. Other descriptive analyses of four summated indices are shown in Table 1.

### Relationship between patient personal and contextual characteristics and four summated indices of patient satisfaction with the physical environments in the waiting areas

The relationships of personal information and contextual factors, and patients' perceptions of the physical environments in the outpatient waiting areas, were examined (see Table 4 and Table 5). Multiple regression analyses revealed that men were statistically more satisfied than women with regard to cleanliness in the physical environment. Older patients were more satisfied with visual and body contact environments. First-time patients were less satisfied with the body contact environment than the returning patients. Outpatients who visited in the morning were more satisfied with the visual environment and cleanliness of the physical environment than those who visited in the afternoon.

**Table 4:** Univariate analyses of patient satisfaction with the physical environments of outpatient waiting areas across patient personal characteristics and visiting information (n = 680)

	**Visual environment**	**Voice environment**	**Body contact environment**	**Cleanliness (overall and restrooms)**	**Analytical techniques**
**Gender**	t = 0.689	t = 0.004	t = -1.357	t = 4.349	t-test
	sig = 0.491	sig = 0.997	sig = 0.175	sig = 0.000***	
				Male patients > Female patients	
**Age**	β = 0.009	β = 0.008	β = 0.009	β = 0.002	Simple regression
	sig0.003**	sig = 0.011*	sig = 0.003**	sig = 0.611	
**Education**	F = 1.520	F = 1.400	F = 3.432	F = 0.306	ANOVA
	sig = 0.208	sig = 0.242	sig = 0.017*	sig = 0.821	
			Patients with undergraduate and above < Patients with elementary and below		
**Visiting frequency**	t = 1.175	t = -0.672	t = -2.827	t = -0.499	t-test
	sig = 0.240	sig = 0.502	sig = 0.005**	sig = 0.618	
			First-time patients < Returning patients		
**Visiting time**	t = 2.520	t = 0.718	t = -1.241	t = 2.073	t-test
	sig = 0.012*	sig = 0.473	sig = 0.215	sig = 0.039*	
	Morning patients > Afternoon patients			Morning patients > Afternoon patients	

Note:

1. Education was measured as four levels: undergraduate and graduate school, senior high school, junior high school, elementary school and below

2. Gender was measured as two levels: male vs. female.

3. Visiting frequency was measured as two levels: first-time patients vs. returning patients.

4. Visiting time was measured as tow levels: morning vs. afternoon

5. Patient salary and living areas (in city residents vs. outside city residents) showed no statistically significant relationships with their perceived physical environments of the medical waiting rooms in the univariate analysis and were not shown in this table.

6. *p < 0.05; **p < 0.01; ***p < 0.001

**Table 5:** Multiple regression analyses of patient satisfaction with the physical environments of outpatient waiting areas across patient personal characteristics and visiting information (n = 680)

	**Visual environment**	**Hearing environment**	**Body contact environment**	**Cleanliness (overall and restrooms)**
**Constant**	-0.207	-0.319	-0.527	0.322
**Gender **(default: male)				
Female	-0.040	0.005	0.142	-0.354***
**Age**	0.008*	0.007	0.007*	-0.001
**Education **(default: Undergraduate and above)				
Senior high school	0.105	0.129	0.085	0.035
Junior high school	-0.015	0.010	0.089	0.095
Elementary school and below	0.104	0.134	0.474	0.244
**Visiting frequency **(default: First-visit patients)				
Returning patients	-0.116	0.058	0.292**	-0.015
**Visiting time **(default: Morning)				
Afternoon	-0.173*	-0.018	0.114	-0.162*

Note:

1. Patient salary and living areas (in city residents vs. outside city residents) showed no statistically significant relationships with their perceived physical environments of the medical waiting rooms in the univariate analysis and were not included in the multiple regression analysis.

2. *p < 0.05; **p < 0.01; ***p < 0.001

In addition, approximately 40% of the respondents recommended enhancing the volume of readings, including newspapers, magazines, and so on. About 32.4% proposed to install the wall-mounted televisions. Also, a few respondents articulated that water (20%), health education brochures (15%), access to wheel chairs (4.4%), and even no-interrupted space for the minority (12%) should be provided.

## Discussion

This study explores how outpatients perceive the physical environments of the waiting areas in a medical center. All the 15 analyzed items were ranked as relatively high with a range of 3.362 to 4.010; environmental cleanliness was the most satisfactory whereas noise level was the least satisfactory.

We also analyzed the relationship between patients' demographics and perceptions of the physical environments of waiting areas, applying the summated indices of patient satisfaction with physical environments in the waiting areas. What was determined is that women were less satisfied with the cleanliness of the physical environments, measured in terms of the holistic and restrooms' surroundings. Traditionally, women take more responsibility for environmental cleanliness at home, which might account for and translate into their having higher expectations of cleanliness than men. Furthermore, in terms of restroom environment, trash tends to accumulate much faster in women's restrooms than in men's restrooms; curiously, both are cleaned at equal intervals in the studied medical center. We suggest that the hospital housekeeping staff check and clean the restrooms more frequently to sustain higher comfort levels for female patient use.

In this study, we determined that older patients were more satisfied with several dimensions of the physical environment, including visual and body contact conditions, than the younger patients. Previous studies on patient satisfaction have shown that patients' age, in an upward direction, is positively related to patient satisfaction [30-32,38-42]. In addition, we found that first-time outpatients registered less favorable perceptions than returning outpatients in body contact environment; these were measured as common components of chair sufficiency and comfort, air freshness, and room temperature. This finding may exist because this medical center has the largest volume of outpatients in central Taiwan. The crowded conditions might surprise first-time visitors, especially those who are used to visiting other healthcare facilities with lower service volumes, leading to uncomfortable feelings in the surroundings, including possible chair insufficiency, odorous air quality, and uncomfortable temperature (i.e. too hot or cold).

Patients' perceptions of the visual environment and cleanliness differed significantly amongst outpatients who arrived in the morning and outpatients who arrived in the afternoon. In this medical center, more physician offices were open, and there was higher outpatient volume in the mornings than in the afternoons (31.67 visits per office in the morning vs. 22.37 visits per office in the afternoon). The researchers noticed that overall lighting was brighter in the morning and slightly reduced in the afternoon, as several physicians' offices were not open and several lighting systems were deactivated. These visual conditions might indirectly influence patients' perceptions of the visual feelings as a whole. We suggest that the hospital continuously maintain the lighting systems in the waiting areas or centralize the waiting areas when some offices are closed and patient volume is lower; these actions may render patients less lonely or afraid. Moreover, cleanliness was perceived as being better in the morning and worse in the afternoon. People perceived the cleanliness (holistic and restrooms' surroundings) based on various factors and even users' customs. Therefore, we suggest that the housekeepers check the holistic environment and the specific areas (i.e., restrooms) more often to better recognize the special needs of afternoon patients.

Certain limitations of this study should also be pointed out. First, all the assessments measured were very standardized so that they could be compared easily across overall patient characteristics; yet, we also provided an open item for the respondents free to respond. A more dynamic and customized evaluation would have been more effective for evaluating patients' demands. For example, our respondents expected the providers to enhance the volume of readings, wall-mounted televisions, health education brochures, water, access to wheel chairs, and no-interrupted space for the minority populations.

A second limitation to this study is that we did not record and ask how long the respondents waited before receiving the questionnaire. It is indeed an important point whether the respondents had sufficient time to appraise the waiting areas. One method we used to overcome this possible pitfall was adding "not applicable" for all individual question items, in case the respondent had no experience with the individual items. In addition, the issue of social desirability bias needs to be mentioned, because some evidence has indicated that patients completing patient satisfaction questionnaires via face-to-face have higher levels of satisfaction as compared to those who receive questionnaires via post [30].

Moreover, another limitation is that our data were collected from only one medical center. A larger sample size comprised of outpatients from different medical centers should be examined to validate the findings from this study. In future research studies involving this context, patient expectations should be examined to provide more information for healthcare managers, a method by which the managers can better design healing environments. In addition, patient health status [43,44] and personality [45] should also be considered to decrease their possible confounding effects in the study of patients' perceptions.

## Conclusion

Many studies have explored how outpatients perceived satisfaction for outpatient services from different dimensions, such as waiting times, courtesy and interpersonal skills, professionalism, and so on. However, few studies have focused on how the atmospherics of waiting areas are associated with outpatient satisfaction.

In this study of outpatients' perceptions of the physical environment of waiting areas, there is still room for improvement via customizing patients' specific characteristics and demands. In addition to evaluating various dimensions of the physical environment, we also examined the effects that outpatient socio-demographics and visiting backgrounds had on patient satisfaction with respect to the physical environment of waiting areas. Gender, age, visiting frequency, and visiting time were all related to patient satisfaction. Furthermore, these factors should be considered when redesigning more comfortable and customized medical care environments in the future.

## Competing interests

The author(s) declare that they have no competing interests.

## Authors' contributions

BYJL designed and conducted this study. CYT coordinated the survey process in the studied medical center. All the authors were involved in the questionnaire development and data analysis, and the manuscript was finally done by BYJL and reviewed by all other authors. GMB refined and edited the content for accuracy and legibility purposes.

## Pre-publication history

The pre-publication history for this paper can be accessed here:


